# Global Analysis of the Specificities and Targets of Endoribonucleases from Escherichia coli Toxin-Antitoxin Systems

**DOI:** 10.1128/mBio.02012-21

**Published:** 2021-09-21

**Authors:** Peter H. Culviner, Isabel Nocedal, Sarah M. Fortune, Michael T. Laub

**Affiliations:** a Department of Immunology and Infectious Diseases, Harvard T.H. Chan School of Public Health, Boston, Massachusetts, USA; b Department of Biology, Massachusetts Institute of Technology, Cambridge, Massachusetts, USA; c Howard Hughes Medical Institute, Massachusetts Institute of Technology, Cambridge, Massachusetts, USA; The Ohio State University

**Keywords:** *Escherichia coli*, RNA-seq, endoribonucleases, toxin-antitoxin systems

## Abstract

Toxin-antitoxin systems are widely distributed genetic modules typically featuring toxins that can inhibit bacterial growth and antitoxins that can reverse inhibition. Although Escherichia coli encodes 11 toxins with known or putative endoribonuclease activity, the targets of most of these toxins remain poorly characterized. Using a new RNA sequencing (RNA-seq) pipeline that enables the mapping and quantification of RNA cleavage with single-nucleotide resolution, we characterized the targets and specificities of 9 endoribonuclease toxins from E. coli. We found that these toxins use low-information cleavage motifs to cut a significant proportion of mRNAs in E. coli, but not tRNAs or the rRNAs from mature ribosomes. However, all the toxins, including those that are ribosome dependent and cleave only translated RNA, inhibit ribosome biogenesis. This inhibition likely results from the cleavage of ribosomal protein transcripts, which disrupts the stoichiometry and biogenesis of new ribosomes and causes the accumulation of aberrant ribosome precursors. Collectively, our results provide a comprehensive, global analysis of endoribonuclease-based toxin-antitoxin systems in E. coli and support the conclusion that, despite their diversity, each disrupts translation and ribosome biogenesis.

## INTRODUCTION

Toxin-antitoxin (TA) systems are genetic modules distributed across bacteria and archaea that can regulate or inhibit the growth of their host cell (1–3). Although first characterized as plasmid maintenance systems, most TA systems are encoded on bacterial chromosomes. There are multiple types of TA systems, classified based on the nature of the antitoxin. One of the most common types is type II TA systems. These encode a toxin that inhibits growth and a coexpressed antitoxin protein that binds and inhibits the toxin. Under conditions that remain poorly defined, antitoxins are thought to be degraded or liberated from their cognate toxins, thereby freeing active toxin to inhibit growth (1). The toxins of TA systems comprise several different protein families, with a diversity of mechanisms for inhibiting cell growth (2, 4).

TA systems have been suggested to promote adaptation to various stresses (3). The ectopic expression of many toxins causes cells to enter a growth-arrested state in which they are stress and antibiotic tolerant (5–7). Additionally, the transcription of many TA systems increases in response to a range of stresses (8). However, there are few cases of strong, reproducible deletion phenotypes for TA systems, and recent work has demonstrated that toxins may not be activated even if their transcription is induced by a stress condition (9, 10). TA systems were also suggested to contribute to spontaneous persister cell formation, but at least for Escherichia coli, this has been refuted (9, 11). Some TA systems are activated during phage infection, with the toxin leading to abortive infection, either by inhibiting host cell processes or by disrupting phage replication or maturation (12–14).

Many bacteria encode endoribonuclease toxins that have been shown to cleave a variety of RNAs, including mRNAs, tRNAs, and rRNAs (15–20). These endoribonucleases can be ribosome independent or ribosome dependent, with the latter class requiring an interaction with the ribosome to reposition catalytic amino acids that then drive cleavage of translated mRNAs, often at a particular position within codons (16, 21, 22). E. coli MG1655 (referred to here as E. coli for simplicity) encodes 11 type II TA systems where the toxin is known or predicted to have endoribonuclease activity, but the precise targets and specificities of most of these toxins remain unclear (Table 1). Six of these toxins have been suggested to be ribosome dependent and thus require translation of their target RNAs.

**TABLE 1:** Summary of previously published data on toxin specificity

Toxin	Family or superfamily	Ribosome dependence	Core cleavage specificity^*a*^
MazF	MazF (4)	No (19, 39)	^ACA[AUC] (19, 39)
ChpB	MazF (4, 40)	No (40)	^A^C[UGC] (40)
RnlA	RnlA, sometimes fused to RNase HI domains (41)	Unknown (41)	Poorly defined (41)
HicA	HicA (27)	No (27)	Poorly defined (27)
MqsR	RelE (structural homology) (31, 32), GinB (4)	No (17, 20, 42), possible ribosome interaction (32)	G^CU (17, 20, 42)
RelE	RelE (4)	Yes (16)	C^G (16); coding frame preference, 12^3 (16)
YhaV	RelE (43)	Yes (44), No (43)	Poorly defined (44)
HigB	RelE (4)	Yes (31)	Poorly defined (31)
YoeB	RelE (4)	Yes (45)	^[AG] (46)
YafO	YafO (4), RelE (weak sequence and structural homology) (31)	Yes (31)	Poorly defined (31)
YafQ	RelE (4)	Yes (47, 48)	AA^A (48), ^A (47); coding frame preference 12^3 (47, 48)

a A ^ indicates site of cleavage; brackets indicate motif may include any of the listed nucleotides at that site.

Previously, we developed a quantitative, paired-end RNA-seq-based approach to systematically map the RNA targets of E. coli MazF, a ribosome-independent toxin. Inducing MazF leads to rapid, widespread cleavage of mRNAs, producing a global disruption in translation, consistent with earlier studies concluding that MazF is an mRNA interferase (19, 23). Additionally, we found that MazF drives the accumulation of rRNA precursors, likely by directly cleaving nascent rRNA and by cleaving ribosomal protein transcripts to prevent their translation, leading to defects in rRNA processing and ribosome assembly. These results suggested that MazF can inhibit cell growth by targeting many mRNAs and inhibiting the proper synthesis of ribosomes. This mechanism of growth inhibition is enabled in part by MazF’s highly abundant cleavage site, the trinucleotide ACA, with some additional specificity in the two nucleotides on either side of the ACA (19). Like MazF, the toxin MqsR also directly cleaves rRNA precursors (20). Whether other toxins are also able to disrupt ribosome biogenesis has, to our knowledge, not been systematically investigated.

To characterize the endoribonuclease toxins in E. coli and compare their specificities and targets, we developed a new RNA-seq pipeline that enabled the high-throughput identification and quantification of RNA cleavage events with single-nucleotide resolution. We found that each toxin recognizes a short, low-complexity motif and, consequently, cleaved much of the transcriptome after induction. Ribosome-independent toxins show no bias toward cleaving a particular codon position and generally cleave throughout the coding region of target transcripts. In contrast, ribosome-dependent toxins showed a clear bias toward cleaving near the 5′ ends of translated mRNAs and at specific locations within subcodons. Our results support the ribosome dependency suggested previously for most toxins but suggest a reconsideration of the ribosome dependency of HicA, YafO, and MqsR. For all the toxins, we found no evidence for the direct cleavage of mature rRNAs or tRNAs. However, each inhibited ribosome biogenesis, leading to the accumulation of abnormal rRNA precursors. Importantly, this inhibition of ribosome biogenesis occurred even for ribosome-dependent toxins that cannot cut rRNA precursors directly, strongly favoring the model that toxins indirectly disrupt ribosome biogenesis through decreased translation of ribosomal protein transcripts. These results suggest that through short, low-information-content motifs, each endoribonuclease toxin in E. coli can efficiently disrupt the proper synthesis of ribosomes and likely other large multiprotein complexes to inhibit growth.

## RESULTS

### Most toxins inhibit cell growth and can be antagonized by a cognate antitoxin.

**FIG 1:**
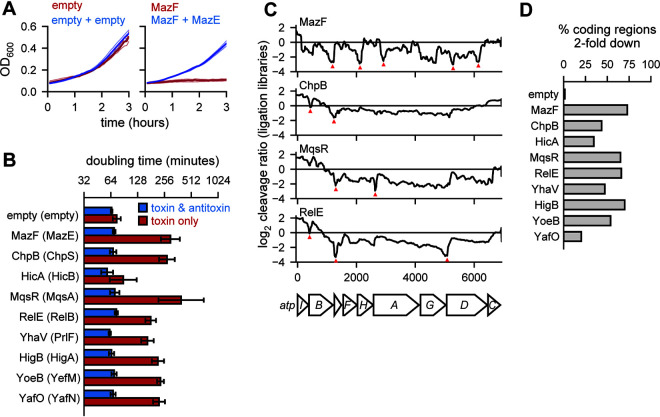
Endoribonuclease toxins inhibit cell growth and broadly cleave the transcriptome. (A) Sample growth data for cells harboring two empty vectors (left) or toxin (MazF)- and antitoxin (MazE)-containing vectors. Toxin was induced (red) or toxin and antitoxin were coinduced (blue) at time zero. Three replicates (faded lines) were used to calculate the mean (solid lines). (B) Doubling times calculated from growth curves like those in panel A using data between 30 min and 2 h for cells expressing a given toxin (red) or toxin and antitoxin (blue). Error bars show the standard deviation of 3 replicates. (C) Cleavage profiles of the highly expressed *atpI-C* region are plotted for four toxins. Selected narrow valleys are highlighted with red triangles. All toxins were expressed for 10 min with the exception of MazF, which was expressed for 5 min. Data shown are from a single ligation RNA-seq library for each toxin compared to a single empty vector library. (D) The percentage of highly expressed (all positions with ≥64 reads in the vector control) coding regions in E. coli with at least one position having a cleavage ratio below −1, or a relative 2-fold downregulation of RNA abundance at the site, after expression of each toxin indicated. Data are from ligation libraries, as in panel C.

To compare the effects of E. coli’s toxins on the transcriptome, we first cloned each toxin and antitoxin into a common expression system. Using separate but compatible low-copy-number plasmids in a wild-type E. coli MG1655 background, we expressed each toxin from an arabinose-inducible promoter and each cognate antitoxin from a tetracycline-inducible promoter. Strains harboring each toxin-antitoxin pair were grown to mid-exponential phase (optical density at 600 nm [OD_600_], ∼0.25 to 0.3) in M9 glycerol, followed by back dilution to an OD_600_ of ∼0.1 with the addition of either arabinose or arabinose and anhydrotetracycline to induce the toxin alone or the toxin and antitoxin, respectively (Fig. 1A). Growth from 30 min to 2 h after induction was used to measure the growth rate. For 8 of 11 TA systems, we observed a >2-fold increase in doubling time when inducing the toxin alone compared to both the toxin and antitoxin (Fig. 1B). For HicA, there was a small but not significant change in doubling time when the toxin was expressed alone, possibly because leaky expression of the antitoxin was sufficient to largely neutralize the toxin. For YafQ and RnlA, induction of the toxin alone did not substantially increase doubling time (Fig. S1A). Because these experiments were conducted in a wild-type background, the endogenous copies of antitoxin (*dinJ* and *rnlB*, respectively) may have been sufficient to inhibit toxicity (13). In subsequent analyses, we focused on the 9 systems (Fig. 1B) where the toxin alone affected growth rate and the antitoxin could rescue the growth defect.

10.1128/mBio.02012-21.1 FIG S1 Toxin expression and method development. (A) Experiment assessing the toxicity of RnlA and YafQ as in Fig. 1B. Doubling times were calculated using time from 90 to 180 min after induction. Bars show a single representative sample. (B) Mapping the cleavage profiles surrounding the top 239 5′-end ratios following 5 minutes of MazF expression. The average 5′-end ratio at these peaks is shown (top). The motif surrounding these peaks is shown (top, inset). The mean cleavage ratio from the fragmented template switch library and ligation library shown for the region surrounding the top 239 5′-end ratio peaks. (C) Comparison of coding region minimum cleavage ratios using template switch libraries and ligation libraries. The *R*^2^ value and number of genes meeting the threshold are plotted across a range of minimum log_2_ expression values (left). Scatterplots of minimum cleavage ratio values using both methods are also shown (right). Download FIG S1, TIF file, 1.4 MB. Copyright © 2021 Culviner et al. 2021 Culviner et al. https://creativecommons.org/licenses/by/4.0/ This content is distributed under the terms of the Creative Commons Attribution 4.0 International license.

### Toxin expression results in widespread degradation of E. coli transcripts.

To determine how toxins affect E. coli mRNAs, we conducted strand-specific paired-end transcriptome sequencing (RNA-seq) after expressing each toxin for 10 min and mapped the full nucleotide coverage of the resulting reads. As in our prior study of MazF (19), we calculated a cleavage ratio (log_2_ + toxin reads/empty-vector reads) at each nucleotide across transcripts. A negative cleavage ratio indicates that a region is either cleaved or destabilized following induction of a given toxin. We examined cleavage ratios across transcripts, here called cleavage profiles (Fig. 1C). Endoribonuclease activity generates valleys within cleavage profiles, whereas changes in general expression or stability cause a transcript's entire cleavage profile to increase or decrease. Visual inspection of the cleavage profiles for the toxins revealed several patterns: (i) the 9 toxins each generated discrete valleys within some transcripts, supporting the notion that each has endoribonucleolytic activity; (ii) cleavage profiles, and the position of the valleys, differed depending on which toxin was induced, implying that the toxins have different cleavage specificities; and (iii) cleavage ratio values often decreased well below 0, indicating that toxin expression led to substantial amounts of cleavage. Apart from YafO and HicA, toxin expression led to a minimum cleavage ratio less than −1 (i.e., >2-fold down) in more than 40% of all expressed coding regions (Fig. 1D). Together, these observations support the conclusion that toxin expression results in a major remodeling of the E. coli transcriptome through direct cleavage of mRNA at a wide variety of sites.

### Mapping and quantification of endoribonuclease-dependent cleavage.

**FIG 2:**
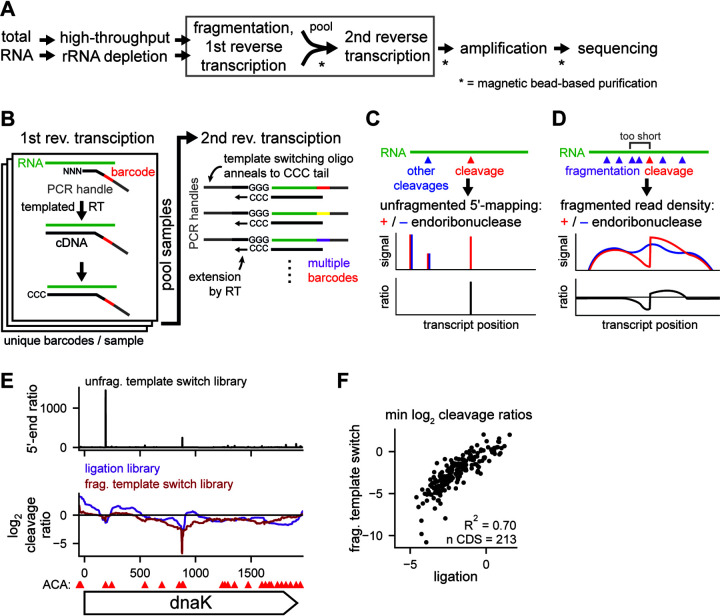
Using template switch libraries to characterize endoribonuclease cleavage. (A) Workflow for template switching libraries. Steps with multiple arrows between are unpooled, and single-arrow steps are pooled. Steps requiring a purification are starred. Steps shown in panel B are boxed. (B) Cartoon illustrating the molecular details of the first and second reverse transcription steps in panel A. (C) Cartoon illustrating how 5′ ends are mapped at single-nucleotide resolution using template switch libraries. “Cleavage” indicates cleavage by a given toxin; “other cleavages” refers to cleavage by housekeeping and other RNases. (D) Cartoon illustrating how the extent of cleavage is quantified using fragmented template switch libraries. (E) Mapping of MazF cleavage across the *dnaK* gene after 5 min of toxin expression. (Top) 5′-end ratios from template switch libraries (geometric mean of 2 replicates). (Middle) Cleavage profiles from fragmented template switching libraries (red; *n* = 2) and ligation libraries (purple; *n* = 2). (Bottom) ACA sites within *dnaK* are shown as red triangles above the gene. (F) Scatterplot comparing coding region minimum cleavage ratios from template switch and ligation libraries within highly expressed coding regions (≥256 reads at all positions in both library types).

To better understand the effect of each toxin on the transcriptome, we initially attempted to extract cleavage specificity from cleavage valleys using an approach analogous to our previous work on MazF (19). However, this approach was complicated by the broad and shallow valleys generated by many of the other toxins compared to MazF (Fig. 1C); as the size of a valley increases, it becomes difficult to identify common sequences between valleys and thus determine a sequence specificity. Other methods, based on enrichment and sequencing of RNAs containing the 5′-OH left by many endoribonuclease toxins, can pinpoint cleavage sites but are not quantitative (18). We therefore developed a dual library construction protocol to achieve both single-nucleotide resolution of cleavage sites and quantification of the extent of cleavage, while also increasing the throughput of library construction compared to prior approaches (Fig. 2A). Briefly, to identify 5′ ends produced from cleavage events, we used random primers containing a sample-specific barcode and PCR handle at their 5′ ends to initiate cDNA synthesis using a Molony murine leukemia virus (MMLV)-type reverse transcriptase (RT). Upon transcribing to the end of an RNA molecule, this RT enzyme adds nontemplated cytosines that can then hybridize to the guanosines at the 3′ end of a template-switching oligonucleotide, which encodes a second PCR handle (Fig. 2B). The resulting cDNAs can then be amplified by PCR and sequenced. The overall approach is similar to existing low-input and single-cell sequencing techniques (24, 25). By sequencing with a custom primer matching the template-switching oligonucleotide, we can pinpoint the 5′ end of the RNAs generated by each endoribonuclease toxin (Fig. 2C).

To quantify cleavage, we measured the loss of reads near cleavage sites, as the site of cleavage cannot be crossed by RT during cDNA synthesis. However, because our technique relies on a free 5′ end for an RNA to be detected, 3′ regions of long, intact transcripts would be underrepresented in our sequencing. Thus, to generate 5′ ends throughout transcripts, we fragmented the input RNA prior to library construction. The resulting cleavage profile shows a loss of read density immediately upstream of a cleavage site, but not necessarily downstream, as cleavage by the toxin may also create a stable, sequenceable 5′ end (Fig. 2D). In sum, our new method involves two libraries from the same initial rRNA-depleted sample, one that allows mapping of cleavage sites with single-nucleotide resolution and one that provides quantification of cleavage at each site. For simplicity, we refer to these libraries as unfragmented and fragmented template switch libraries, respectively. The unfragmented template switch library provides information and resolution similar to those of previously reported 5′ mapping methods, while the fragmented template switch library provides information and quantitative measures of cleavage similar to those of our previously developed paired-end RNA-seq approach (18, 19). The template switch-based libraries developed here have the advantage of enabling higher throughout, lower cost, and the simplicity of a single workflow.

To validate this new methodology, we characterized the cleavage sites of MazF, whose cleavage specificity and targets we had mapped in a prior study using the adapter ligation-based, paired-end RNA-seq protocol we used for our initial toxin expression data (Fig. 1C) (19); for simplicity, we refer to this as a ligation library. For both the template switch and ligation libraries, we expressed MazF from a low-copy-number plasmid for 5 min and compared it to an empty vector control. For the representative transcript *dnaK*, MazF cleavage profiles derived from the ligation library identified two cleavage valleys at nucleotide positions ∼200 and ∼900 (Fig. 2E, bottom). The unfragmented template switch library produced two major peaks within *dnaK* at the same two sites (Fig. 2E, top). The corresponding fragmented template switch library produced a cleavage profile with valleys at these two sites (Fig. 2E, bottom). The first site had a minimum cleavage ratio of −1, while the second site had a minimum ratio of −7, suggesting that the second site is more extensively cleaved. Importantly, although the 5′-end ratios suggest that the first site is more strongly cleaved, we previously found that the stability of 5′ ends generated from cleavage events does not necessarily correlate with their degree of cleavage (19).

To broadly compare our new method (Fig. 2C and D) to the prior ligation library method, we identified the peaks with a 5′-end ratio of ≥1,000 in MazF’s unfragmented template switch library (*n* = 239). In the fragmented template switch library and the ligation library, these sites featured a sharp drop in read density upstream (Fig. S1B). From these 239 cleavage sites, we identified a sequence motif almost identical to that generated by the ligation library method (19). Additionally, the minimum cleavage ratio within each highly expressed gene was strongly correlated between the ligation and fragmented template switch libraries (*R*^2^ = 0.70) (Fig. 2F). This correlation decreased as we decreased the expression threshold used, indicating that cleavage is more difficult to quantify for lower-expression genes (Fig. S1C). Taken together, these results indicate that template switching libraries are a rapid and powerful alternative to ligation libraries that can achieve both single-nucleotide resolution of cleavage sites and quantification of RNA cleavage at each site.

### Endoribonuclease toxins cleave mRNAs with limited specificity.

**FIG 3:**
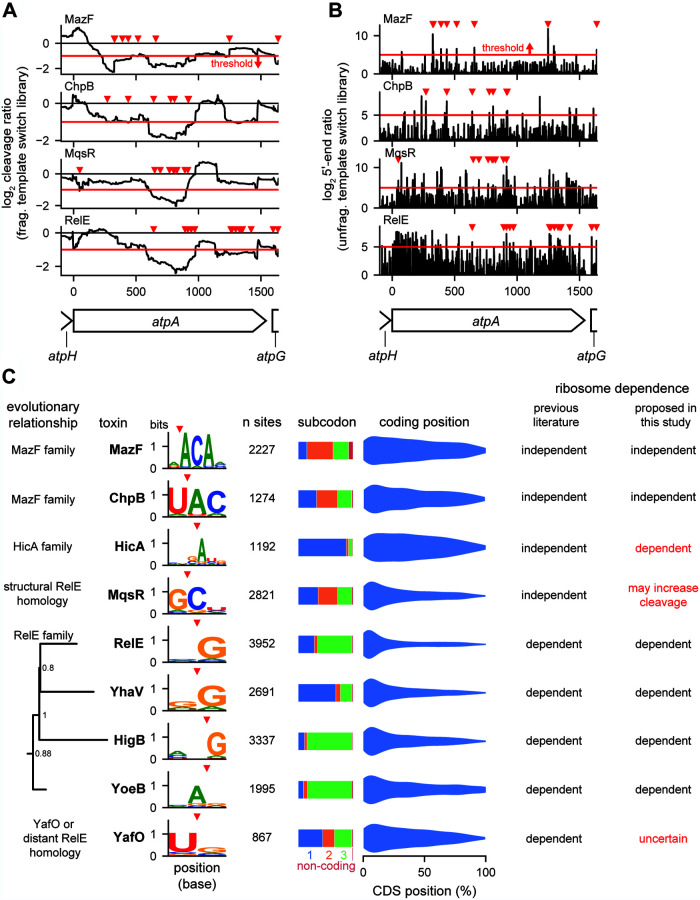
Cleavage specificities of 9 endoribonuclease toxins. (A and B) Cleavage profiles (A) and 5′-end ratios (B) from template switch libraries of the highly expressed *atpA* gene are plotted for the four toxins indicated. Each toxin was expressed for 5 min. Data are means for 2 replicates for each toxin and empty vector control. Peaks that were identified as cleavage sites with the thresholds described in for panel C are marked with red triangles. Thresholds for cleavage ratio and 5′-end ratio are shown as red lines. (C) Summary of cleavage specificities identified for each toxin in expressed non-rRNA regions using a combination of fragmented and unfragmented template switch libraries. Sites (cleavage site plus 10 upstream nucleotides) were required to meet a minimum expression threshold of 64 reads at all positions. Sites were called as peaks if the 5′-end ratio was ≥32 at the cleavage site with a ≤2-fold decrease in read density of the fragmented library in the region upstream of the cleavage site. Nucleotides with ≥0.05 bits of information were included in the motifs shown. Evolution tree of RelE family built using 1,144 protein sequences, with only E. coli sequences shown.

Using our new method, we sought to determine the cleavage specificity of the 9 E. coli endoribonuclease toxins that inhibited growth in our system (Fig. 1B). We expressed each toxin in a wild-type MG1655 background for 5 min from a low-copy-number plasmid and then generated template switching libraries. As in ligation libraries (Fig. 1C), we saw evidence of cleavage across highly expressed regions such as the *atp* operon following expression of all 9 toxins (Fig. 3A); the addition of the 5′-end ratios from the unfragmented template switch library resolved many of these regions to specific sites of cleavage (Fig. 3B). To globally identify and characterize cleavage sites, we selected 5′-end ratio peaks in the unfragmented libraries that had (i) ≥32-fold more signal in the toxin expression sample than the empty vector control and (ii) a log_2_ cleavage ratio less than or equal to −1 in the fragmented library immediately upstream of the peak. Finally, we required regions to have a high level of expression (≥64 reads crossing a given base) in the empty vector sample to ensure that we had enough reads to accurately separate cleavage events from noise in poorly expressed genes. For each toxin, we used the identified cleavage sites (ranging in number from 867 to 3,952) to identify sequence motifs directly at the site of cleavage (Fig. 3C, left). Because some toxins are ribosome dependent, we used the annotated reading frames to identify the subcodon position of cleavage within coding regions (Fig. 3C, middle). Finally, we plotted the density of cleavage events across coding regions in a 5′-to-3′ direction (Fig. 3C, right).

Our data indicate that the 9 E. coli endoribonuclease toxins examined here have relatively weak cleavage specificities (Fig. 3C), consistent with a broad disruption of the transcriptome (Fig. 1D). The motifs identified for MazF, ChpB, MqsR, and RelE partially or completely matched previously reported motifs (Table 1). Consistent with the classification of MazF and ChpB as ribosome-independent nucleases, we found that cleavage was not highly dependent on codon position. Additionally, cleavage by MazF and ChpB was seen throughout the coding region of mRNAs, though with some bias toward the 5′ end, possibly reflecting differences in RNA secondary structure across coding regions or the fact that cleavage toward the 5′ end of a transcript may destabilize the downstream fragment, reducing the number of subsequent cleavage events detected (26). MazF and ChpB are both members of the MazF family of nucleases and are more closely related to each other than the other endoribonucleases in E. coli. Consistent with this relationship and similarity, MazF and ChpB had similar sequence motifs, with MazF cleaving 5′ of an ACA motif and ChpB cleaving immediately before the AC of a UAC motif.

In contrast to MazF and ChpB, and consistent with its classification as a ribosome-dependent nuclease and in agreement with prior literature (16), RelE cleavage showed a high dependence on codon position, with most cleavages immediately before a guanosine in the third position of a codon, with a very strong bias in cleavage toward the 5′ end of mRNAs. The toxins YoeB, YhaV, and HigB, which are members of the RelE family and reported to also be ribosome dependent, also had (i) a skewed cleavage preference within codons and (ii) a preference for the 5′ end of mRNAs. RelE, YhaV, and HigB form a clade, and each favors a G just downstream of the cleavage site, whereas YoeB has specificity driven by an A upstream of the cleavage site. Although MqsR and YafO have sometimes been included as RelE family members, their cleavage specificity and limited codon position preference diverged from those of the core RelE family members.

Three toxins contradicted the general trends noted for the ribosome-independent and ribosome-dependent toxins. HicA was previously proposed to be ribosome independent, as primer extension studies indicated cleavage of tmRNA lacking the codon used to initiate *trans*-translation, but mRNAs with start codons removed were not examined (27). Although we saw cleavage throughout mRNAs, like with MazF and ChpB, HicA had a strong bias for cleaving before the first position in codons, similar to the ribosome-dependent toxins. MqsR has also been proposed to be a ribosome-independent toxin and has been shown to cleave rRNA precursors (17, 20). Though we saw no preference with respect to codon position, supporting its ribosome independence, it had a strong bias toward cleaving the 5′ ends of mRNAs, like ribosome-dependent toxins, indicating that the ribosome may play a greater role in its specificity than in other ribosome-independent toxins. Finally, YafO, which was suggested to be ribosome dependent, had no strong preference with respect to codon position and cleaved throughout mRNAs like the ribosome-independent toxins (28). These results for HicA, MqsR, and YafO should prompt a reconsideration and further investigation of their interaction with ribosomes and translation.

### tRNA is not a direct target of the endoribonuclease toxins in E. coli.

**FIG 4:**
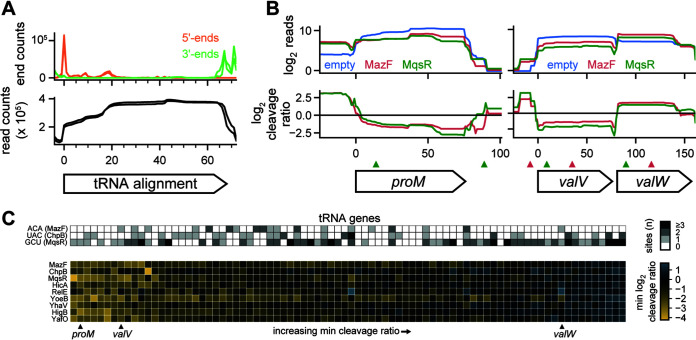
tRNA is not a direct target of E. coli endoribonuclease toxins. (A) 5′ ends (orange) and 3′ ends (green) of fragments from template-switching libraries containing an empty vector (*n* = 2) were mapped to an alignment of 68 tRNA genes (all tRNAs <78 nt in length except *hisR*; top). The total read counts were also plotted (bottom). (B) Read counts (top) and cleavage profiles (bottom) of selected tRNA genes following induction of MazF (red) and MqsR (green) for 5 min compared to the empty vector control. Cleavage sites for MazF (ACA; red) and MqsR (GCU; green) are plotted as triangles above the genes. (C) (Bottom) Heat map of minimum cleavage ratios in well-expressed tRNAs after 5 min of expression of each toxin. (Top) The number of MazF, ChpB, and MqsR cleavage sites within each tRNA is shown as a heat map. The example tRNAs shown in panel B are marked below the heat map.

In other organisms, endoribonuclease toxins, particularly those in the VapC family, have been shown to target tRNA (15). Although E. coli MG1655 encodes no VapC toxins, we wanted to assess whether the 9 toxins of interest here could cleave tRNA. Mature tRNA is difficult to sequence due to a combination of its small size, structure, and nucleotide modifications, so we first verified that a modified template switch library protocol allowing smaller RNA species could capture tRNA sequences with mature 5′ and 3′ ends by mapping tRNA reads to a consensus tRNA alignment (Fig. 4A). Indeed, we observed high read counts across the entire tRNA, with 5′ and 3′ ends corresponding to the expected ends of mature tRNA.

After expression of each toxin, the read counts for individual tRNAs sometimes changed across the entire gene body, but without producing valleys or cliffs in the cleavage ratio, as would be expected for cleavage events (Fig. 4B). For example, the toxins MazF and MqsR each altered the expression profiles of the tRNAs encoded by *proM*, *valV*, and *valW*, but without valleys that coincided with the core MazF and MqsR motifs ACA and GCU, respectively. We expanded this analysis to all highly expressed tRNAs (*n* = 82 of 86), by measuring the minimum cleavage ratio within each tRNA after toxin induction (Fig. 4C). Many tRNAs had cleavage ratios close to 0 and in the cases where there were low cleavage ratios, they did not correlate with the presence of cleavage motifs of MazF, ChpB, and MqsR (ribosome-independent toxins with cleavage motifs of at least 3 nucleotides). Further, the changes in tRNA expression observed were broadly correlated across most toxins, supporting a model in which the endoribonucleases indirectly alter the expression, maturation, or recycling of a subset of tRNAs. Taken together, our results show no evidence of direct tRNA cleavage by E. coli toxins but identify common changes in tRNA levels.

### Mature ribosomes are not a target of E. coli’s endoribonuclease toxins.

**FIG 5:**
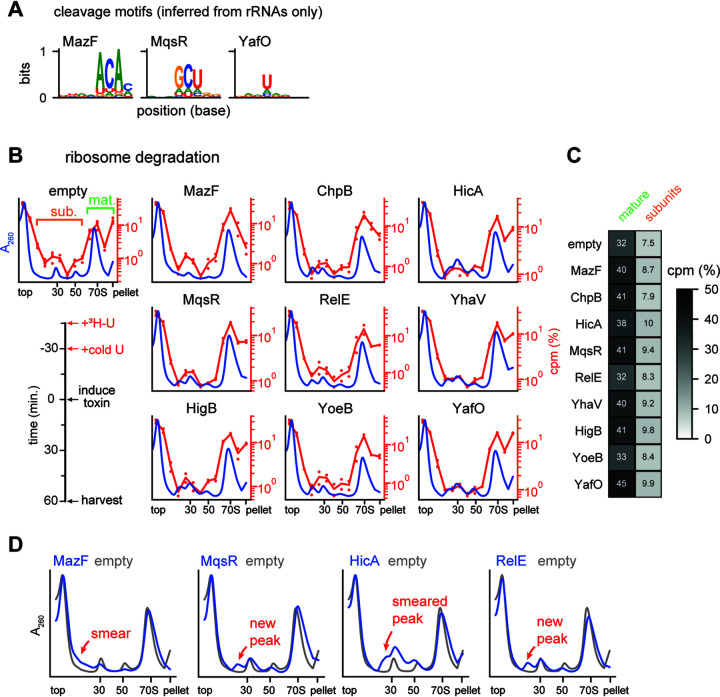
Mature ribosomes are not a target of toxins. (A) Cleavage motifs identified from the peaks within rRNA and rRNA precursor regions after 30 min of toxin expression. Toxins broadly matching the motif defined in Fig. 3C are shown here, with toxins that did not match shown in Fig. S2A. (B) Sucrose gradients showing effect of toxin expression on mature ribosomes. The timeline of the experiment is shown on bottom left. Average *A*_260_ values are plotted in blue (empty vector, *n* = 8; each toxin, *n* = 4). Numbers of counts per minute for ^3^H are plotted on the right axis as a percentage of total signal (empty vector, *n* = 4; each toxin, *n* = 2); dots show the values for each replicate, with lines showing averages. (C) Summed ^3^H signal from the mature ribosome and precursor fractions from panel B. Mature and subunit fractions are defined on the empty vector sample in panel B. (D) Average *A*_260_ values for selected toxins compared to the empty vector sample from panel B are enlarged relative to panel B to highlight changes from toxin expression. The location of smears and peaks relative to the empty vector are highlighted.

Previous work has indicated that MazF family toxins can target rRNAs (18, 29), although recent studies of E. coli MazF and MqsR have shown that these toxins primarily target rRNA precursors (19, 20). To determine if we could identify likely cleavage sites on the rRNA for any other toxins, we generated unfragmented template switch libraries following 30 min of toxin expression without ribosome depletion. We then took, using only rRNA regions, the top 30 5′-end ratio peaks and assessed whether the cleavage specificity derived from these peaks matched that found in the wider transcriptome (Fig. 5A; Fig. S2A). These peaks from rRNAs matched the characterized cleavage specificity only in the cases of MazF, MqsR, and YafO, suggesting that rRNA cleavage by these toxins may be direct. Visual inspection of top cleavage peaks generated by MazF and MqsR showed strong signals unique to each toxin (Fig. S2B); YafO peaks were much weaker relative to background, suggesting that if cleavage does occur, rRNA is not a common target. Together, these results reaffirm that MazF and MqsR are capable of cleaving rRNA (19, 20) and suggest that other toxins, with the possible exception of YafO, do not directly cleave rRNA.

10.1128/mBio.02012-21.2 FIG S2 Toxin expression and ribosome cleavage. (A) Apparent cleavage motifs identified from rRNA regions for remaining toxins as described in Fig. 5A. (B) Selected peaks from top 5′-end ratios (geometric mean of *n* = 2) used to define the motifs for MazF, MqsR, and YafO. Peaks are shown with red triangles, and the surrounding 8 nucleotides are shown. Nucleotides matching the core motif are highlighted in red. Download FIG S2, TIF file, 1.3 MB. Copyright © 2021 Culviner et al. 2021 Culviner et al. https://creativecommons.org/licenses/by/4.0/ This content is distributed under the terms of the Creative Commons Attribution 4.0 International license.

To better assess whether induction of each endoribonuclease toxin affects mature rRNA, either directly or indirectly, we added [^3^H]uridine to exponential-phase cells to label rRNA, chased with unlabeled uridine, induced toxin, and then harvested cells to examine polysomes (Fig. 5B, bottom left). This scheme allowed us to assess whether each toxin affected mature, labeled ribosomes. We used sucrose density gradients to separate polysomes (in the pellet), monosomes, and ribosomal subunits/precursors (Fig. 5B). The tritium signal in the mature ribosome fractions was similar in cells expressing toxin and those harboring an empty vector, and there was no substantial increase in tritium signal in subunit/precursor fractions for cells expressing toxin (Fig. 5C). These results suggest that the toxins do not drive the degradation or disruption of mature ribosomes or strongly affect the available pool of ribosomes. We conclude that mature ribosome degradation is unlikely to be a major contributor to toxin-dependent growth arrest.

### Endoribonuclease toxins inhibit ribosome biogenesis.

After inducing each toxin in the labeling experiment described above (Fig. 5B), we noticed the appearance of new peaks and smears near the 30S and 50S peaks in the *A*_260_ traces from our sucrose gradients (Fig. 5D). These features were subtle but highly reproducible and varied between toxins. Because we saw no significant loss in the signal corresponding to mature ribosomes, we inferred that these new peaks arose from disruptions to rRNA synthesis or maturation.

**FIG 6:**
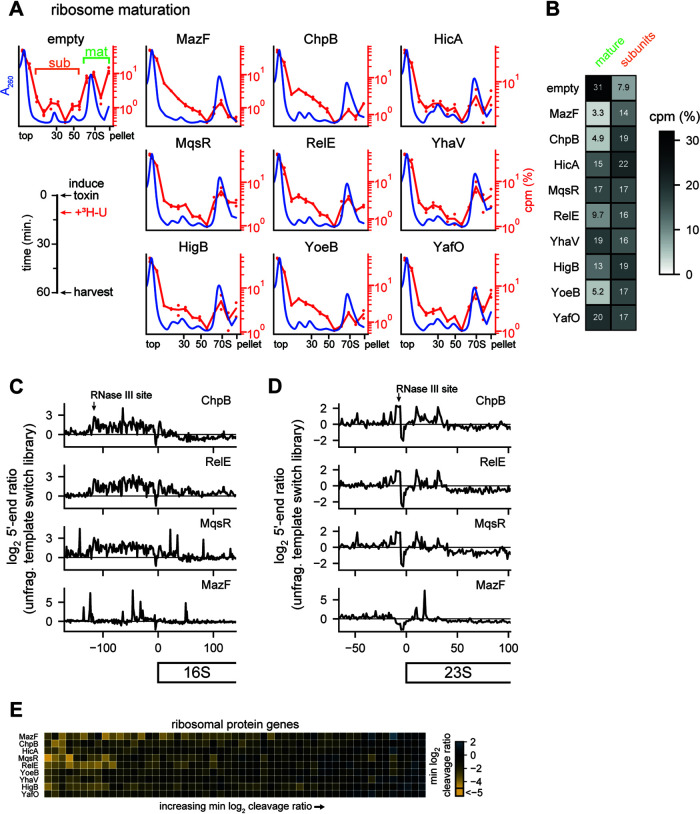
Toxins disrupt ribosome biogenesis. (A) Sucrose gradients showing the effect of toxin expression on ribosome biogenesis. The timeline of the experiment is shown on bottom left. Average *A*_260_ values are plotted in blue (empty vector, *n* = 8; each toxin, *n* = 4). The numbers of counts per minute for ^3^H are plotted on the right axis as a percentage of total signal (empty vector, *n* = 4; toxin, *n* = 2); dots show the values for each replicate, with lines showing the averages. (B) Summed ^3^H signal from the mature ribosome and precursor fractions from panel A. Mature and subunit fractions are defined on the empty vector sample in panel A. (C) Log_2_ 5′-end ratios for four toxins after 30 min of expression data are the means for two replicates each of toxin and empty-vector samples. The site of RNase III maturation is labeled in the top plot. (D) The 5′-end ratios of the 23S loci plotted as in panel C. (E) Heat map of minimum cleavage ratios for well-expressed (≥64 reads at all positions) ribosomal protein genes from fragmented template switch libraries for each toxin after 5 min of expression. Data are means for two replicates for each toxin and empty vector.

To assess how each toxin impacted the biogenesis of new ribosomes, we induced toxins for 10 min and then added [^3^H]uridine without chasing, with cells harvested after an additional 50 min of growth (Fig. 6A, left). As described above, we used sucrose density gradients to separate polysomes (in the pellet), monosomes, and ribosomal subunits (Fig. 6A). Relative to an empty vector control, expression of each toxin now substantially increased the incorporation of tritium into the region between the top of the gradient and the monosome peak (Fig. 6B). Concomitantly, each toxin also reduced the incorporation of radiolabel into mature ribosomes relative to the empty vector control. Broadly, the fractions containing tritium signal in this experiment were coincident with both the normal subunit peaks observed in the empty vector sample and the aberrant peaks and smears associated with toxin expression (Fig. 5D and 6A). Together, these findings indicate that the 9 endoribonuclease toxins examined each disrupt rRNA biogenesis, leading to the accumulation of aberrant rRNA precursors.

To gather additional evidence, we examined our 5′-end mapping data from the unfragmented template switch libraries, focusing specifically on the 16S and 23S rRNAs after 30 min of toxin expression. Here, we found common patterns across all toxins with the exception of MazF. Unlike in mRNAs, where toxins generate clear peaks in the 5′-end ratio (Fig. 3B), we observed a broad increase in the 5′-end ratio between the RNase III maturation site and the 5′ end of mature 16S rRNA (Fig. 6C); this increase in immature ends supports our conclusion that the new peaks observed on sucrose gradients resulted from improper maturation of rRNA (Fig. 6A). The 23S rRNA also showed evidence of aberrant precursors; here, there was an increase in 5′-end ratio directly at the RNase III maturation site, again indicating a backlog of unprocessed precursors (Fig. 6D). In contrast to the other toxins, MazF’s 5′-end ratio in both regions was dominated by tall peaks like those we observed during cleavage of mRNA (Fig. 3B). MazF’s sucrose gradients were also unique compared to those of the other toxins. While other toxins produced new peaks or peak shoulders near the 30S and 50S, MazF instead decreased the intensity of the two subunit peaks and caused the incorporation of tritium primarily near the top of the sucrose gradient (Fig. 5D and 6A). Taken together, these results suggest that MazF may be unique in its strong, direct cleavage of ribosomal precursors and that other toxins, including the other ribosome-independent toxins, may inhibit ribosome biogenesis indirectly.

Notably, the same patterns and effects were seen with both ribosome-dependent and ribosome-independent toxins. As the ribosome-dependent toxins like RelE do not directly cleave rRNAs and almost exclusively target translated mRNAs, we conclude that these toxins primarily inhibit ribosome biogenesis by cleaving ribosomal protein transcripts. Thus, our results suggest that these toxins’ inhibition of ribosome biogenesis is likely through inhibition of ribosomal protein synthesis. Since ribosomal proteins are bound while rRNA is synthesized, insufficient ribosomal proteins can lead to defects in folding and proper rRNA maturation (30). Expression of endoribonuclease toxins have long been associated with decreases in bulk translation, and, using ribosome profiling on cells expressing MazF, we previously linked cleavage sites with “traffic jams” of stuck ribosomes and local translation inhibition (19). Thus, to determine if ribosomal protein synthesis is inhibited by each toxin’s expression, we measured the cleavage profile minimums in ribosomal protein coding regions (Fig. 6E). We observed that toxins inhibit a unique set of ribosomal proteins. The unique set of proteins targeted may lead to different blocks in ribosome maturation and drive the different but reproducible aberrant precursor peaks observed on expression of each toxin. Taken together, our observations suggest that each toxin creates a set of ribosome precursors incapable of efficiently or properly maturing into normal 70S particles. Because translation-dependent toxins also produce such precursors, we favor a model in which an improper ratio of ribosomal proteins unique to each toxin’s cleavage specificity leads to a defect in ribosome biogenesis, likely as a primary means of suppressing cell growth.

## DISCUSSION

### Toxins degrade the E. coli transcriptome with minimal specificity.

A striking feature of toxin-antitoxin systems is that a single strain of bacteria often encodes multiple systems in which the toxins have similar biochemical activities (9). For endoribonuclease toxins, this observation raises the question of whether individual toxins target the same transcripts to inhibit growth or whether individual toxins have specialized functions or specificities. However, to date there has been no systematic, global characterization of all endoribonuclease-based TA systems from a single organism using the same methodology. To do this, we developed a new sequencing pipeline to enable the high-throughput mapping and quantification of RNA cleavage with single nucleotide resolution. The results are in general agreement with our previous study of MazF and other, independent global characterizations of RelE and MqsR (16, 17, 19). Using our new pipeline, we documented the global patterns of RNA degradation triggered by each of 9 toxins in E. coli (Fig. 3C).

Each toxin had a short, low-complexity cleavage motif, the longest of which was for MazF and ChpB, with high information content at 3 core nucleotides. Notably, differences in nucleotide specificity tracked with evolutionary distance between toxins. Three toxins in our set share clear homology to RelE: YoeB, YhaV, and HigB. Of these, YoeB was the most distantly related both evolutionarily and in terms of its cleavage motif, cleaving after an A whereas the others cleaved before a G. MqsR and YafO have been suggested to share very distant homology to RelE (31, 32), and notably, both had sequence specificity quite divergent from that of RelE, YhaV, and HigB. ChpB and MazF are homologous and relatively closely related. This similarity was reflected in their similar cleavage specificities, with ChpB’s motif effectively adding a U at position −1 and removing an A at position 3 relative to MazF’s motif. We also noticed that the cleavage specificities of some toxins are similar enough that they target some of the same sites (Fig. S3). Assessments of sites that could be cleaved by both MazF and ChpB as well as RelE and HigB showed a greater number of shared sites than would be expected by chance. Given this observation and the generally low complexity of toxins’ motifs, we conclude that toxins are unlikely to specialize in targeting particular transcripts.

10.1128/mBio.02012-21.3 FIG S3 Comparison of toxin cleavage sites and motifs. (A) Comparison of core motifs of MazF and ChpB, with the site of cleavage marked by a red triangle (left). Overlap of cleavage of U^ACA sites by both toxins (right). Cleavage was assessed at sites with ≥64 reads in the empty vector sample. Cleavage was defined as a 5′-end ratio of ≥10 and a cleavage ratio less than or equal to −1 in the fragmented template switch library. Statistical significance of the category “both” was assessed by permuting the classifications “cleaved” and “uncleaved” classifications for each toxin across the sites 100,000 times. (B) Comparison of RelE and HigB as in panel A. Codon position preferences are shown at the top, and RelE’s lower-probability cleavage before subcodon 1 is shown with a greyed-out cleavage motif. Download FIG S3, TIF file, 1.2 MB. Copyright © 2021 Culviner et al. 2021 Culviner et al. https://creativecommons.org/licenses/by/4.0/ This content is distributed under the terms of the Creative Commons Attribution 4.0 International license.

Our results support earlier assignments of MazF, ChpB, and MqsR as ribosome-independent toxins, as none exhibits strong subcodon bias within translated substrates and MazF and MqsR both show evidence of rRNA cleavage. The related toxins RelE, HigB, YhaV, and YoeB each showed both a subcodon bias and a preference for cleaving the 5′ ends of coding regions, supporting the conclusion that each is a ribosome-dependent toxin (16). MqsR has also been suggested to also be a ribosome-dependent toxin (32). In support of this conclusion, we found that MqsR’s cleavage sites were biased toward the 5′ ends of coding regions as with RelE. However, the lack of a subcodon bias and the ability of MqsR to cleave rRNA (Fig. 3C and 5A) suggest that translation is not necessary for MqsR cleavage. MqsR’s cleavage pattern is suggestive that strict classification as ribosome dependent or independent may not be appropriate for some toxins, as the ribosome’s activity may play a role in cleavage specificity even without being strictly required for catalysis. HicA was previously characterized as the defining member of a family of ribosome-independent toxins (27), but it exhibited a strong preference for cleaving immediately before the first position of a codon (Fig. 3C). The strength of this codon specificity is hard to reconcile with a completely ribosome-independent model of HicA cleavage. However, HicA showed less 5′ bias than other ribosome-dependent toxins, which could indicate inefficient ribosome-dependent cleavage or ribosome-independent cleavage. Finally, although YafO was suggested previously to be ribosome dependent for cleavage based on *in vitro* cleavage studies and binding of YafO to 50S ribosome subunits (28, 31), we found that it lacked both a clear subcodon bias and a 5′-end preference (Fig. 3C). Combined with weak signal matching YafO’s cleavage motif from the rRNA (Fig. 5A), our results at least indicate that YafO behaves differently from other ribosome-dependent endoribonucleases. In sum, our results support a reassessment of the role of the ribosome in substrate specificity in RNase toxins, particularly for MqsR, HicA, and YafO.

### Toxins inhibit ribosome biogenesis through cleavage of ribosome protein transcripts.

All of the toxins examined here led to significant disruptions of ribosome biogenesis. We infer that this results primarily from the cleavage of ribosomal protein transcripts, not rRNA directly, as ribosome biogenesis was disrupted even when expressing ribosome-dependent toxins that can only cleave mRNA. Ribosomal proteins bind nascent rRNA as it is transcribed to initiate the maturation of new ribosomes. These ribosomal proteins are required in the correct stoichiometry for efficient folding and maturation of ribosomes, so cleavage of their transcripts leads to the disruptions in ribosome biogenesis documented here (Fig. 6A to D). Intriguingly, different toxins created different “fingerprints” of aberrant ribosome precursors (Fig. 5D). This observation is consistent with the model that blocking the translation of different sets of ribosomal proteins results in different disruptions of ribosome biogenesis. Future analysis of the RNA and protein content of these precursors might shed light on the stages of ribosome biogenesis that are inhibited and reveal whether they are normal intermediates in ribosome maturation. It will also be of interest to determine whether cells in which the relevant antitoxin is restored, and which thus resume growth, are able to redirect these toxin-induced ribosome precursors into mature 70S particles or whether they must first be disassembled or degraded.

### A flexible pipeline for high-throughput bacterial RNA-seq.

In this study, we developed a new RNA-seq pipeline for identifying the cleavage sites of endoribonuclease toxins (Fig. 2A). Combined with improvements to our previously reported rRNA depletion technique (33), the two unpooled steps in this pipeline are both 96-well compatible, and all subsequent purifications are pooled and rely on magnetic beads to save time and material costs. Because our pipeline uses the first RT step and template switching to integrate PCR amplification handles, it removes many enzymes and reaction steps present in traditional protocols and instead uses only reverse transcriptase and a PCR amplification mix. Together, these measures vastly reduced the cost of sequencing library preparation: a set of 24 libraries with this method costs ∼$10 per library (including rRNA depletion) in enzymes and specialized consumables.

Though we used our pipeline primarily to map cleavage sites, the core protocol can be adapted to capture different types of gene expression data and various RNA species. The most obvious application is to improve the feasibility of large-scale studies of bacterial gene expression; when used with a fragmentation step, the technique can quantify changes in gene expression, even at a sub-gene-level resolution (Fig. 2E and F). Further, by modifying the size-selection protocol, we were able to capture mature tRNA ends (Fig. 4A). By pooling early in the pipeline, we needed to conduct only a single gel purification on a barcoded pool of 24 tRNA libraries, making this technique a powerful but simple way to capture tRNA reads. Additionally, we found that our technique also captured 5′ ends arising from native RNA processing during rRNA maturation (Fig. 6C); by combining our approach with enzymatic preprocessing steps already used in other 5′-end mapping protocols (18), it may be possible to rapidly distinguish transcription start sites and various RNA processing events by selectively sequencing RNA with 5′-triphosphate, 5′-monophoshate, or 5′-OH. As a whole, our approach provides a flexible tool for large-scale bacterial RNA-seq studies.

### Concluding remarks.

There are an estimated 25,000 ribosomes per cell in actively growing E. coli that are capable of translating enough material to double the cell volume every 20 min. By inhibiting ribosome biogenesis without degrading mature ribosomes, the endoribonuclease toxins of toxin-antitoxin systems can drive a rapid cessation of growth, while sparing the massive energy investment already made in mature ribosomes, thus avoiding a major fitness cost associated with toxin activation.

For the 9 toxins described here, we identified very short, low-information cleavage motifs. This limited specificity enables each toxin to drive the degradation of wide swaths of the transcriptome, including ribosomal protein transcripts, leading to a halt in ribosome biosynthesis. However, without knowing the conditions under which toxin-antitoxin systems are normally activated, it remains unclear if ribosome maturation and growth inhibition are their primary target and function, respectively. In principle, endonuclease toxins could inhibit any cellular process requiring synthesis of a protein whose RNA encodes a cleavage site; given their short motifs, the likelihood of inhibiting a process requiring the synthesis of multiple proteins is very high. In the case of bacteria infected with bacteriophage, inhibiting the synthesis of new phage particles may enable populations of bacteria to slow or stop the spread of infection. Indeed, there is growing evidence that the endoribonuclease activity of toxin-antitoxin systems can both be activated by and defend against bacteriophage (13, 14). Future exploration of the activation conditions of these toxins will shed light on their role in bacterial survival and stress response.

## MATERIALS AND METHODS

### Growth conditions.

Escherichia coli was grown in M9 (10× stock made with 64 g/liter Na_2_HPO_4_·7H_2_O, 15 g/liter KH_2_PO_4_, 2.5 g/liter NaCl, and 5.0 g/liter NH_4_Cl) medium supplemented with 0.1% Casamino Acids, 0.4% glycerol, 2 mM MgSO_4_, and 0.1 mM CaCl_2_. During experiments, cells were grown at 37°C in either a rotor drum (overnight cultures), an orbital shaker at 200 rpm (bulk cultures for ribosome maturation and degradation), or a Synergy H1 plate reader (BioTek) in 24-well plates using double orbital rotation at 307 cpm (sequencing libraries and toxicity/rescue). All experiments were started from individual colonies following growth overnight on LB (10 g/liter NaCl, 10 g/liter tryptone, 5 g/liter yeast extract) agar plates; colonies were grown overnight in M9 medium. Antibiotics were used at the following concentrations (liquid/plates): carbenicillin, 50 μg ml^−1^/100 μg ml^−1^, and chloramphenicol, 20 μg ml^−1^/30 μg ml^−1^.

### Strain and plasmid construction.

Modified pBAD30 plasmids were used for expression of toxins. A sequence containing a ribosome binding site was added between the EcoRI and SacI sites in the MCS of the pBAD plasmid just prior to the toxin’s start codon. Toxin and antitoxin sequences were amplified from the MG1655 E. coli chromosome. Antitoxins were inserted into pKVS45 using Gibson assembly. For some TA systems (HicA, YhaV, MqsR, and HigB), expression levels of toxin and/or antitoxin were altered by changing the 5′ untranslated regions (UTRs) of the encoding plasmids to enable rescuable toxicity. All experiments were conducted in MG1655 harboring the toxin-expressing plasmid or both the toxin- and antitoxin-expressing plasmids. Strains are shown in Table S1.

10.1128/mBio.02012-21.4 TABLE S1 Strains. Download Table S1, DOCX file, 0.01 MB. Copyright © 2021 Culviner et al. 2021 Culviner et al. https://creativecommons.org/licenses/by/4.0/ This content is distributed under the terms of the Creative Commons Attribution 4.0 International license.

### Induction of toxicity and rescue.

To measure toxicity and rescue, individual strains harboring each toxin-antitoxin pair were grown from overnight cultures to mid-exponential phase (OD_600_, ∼0.25 to 0.3) in M9 glycerol followed by back dilution to an OD_600_ of ∼0.1 on a 24-well plate with wells containing either arabinose (0.2% final concentration) or arabinose and anhydrotetracycline (100-ng/ml final concentration) to induce the toxin alone and or the toxin and antitoxin, respectively. Growth was monitored by a plate reader set to read OD_600_ every 5 min.

### RNA extractions.

Cultures (1 ml) were mixed with stop solution (110 μl; 95% ethanol and 5% phenol) and pelleted by centrifugation for 30 s at 13,000 rpm on a tabletop centrifuge. Pellets were flash frozen and stored at −80°C. Cells were lysed by adding TRIzol (Invitrogen) preheated to 65°C directly to pellets, followed by 10 min of shaking at 65°C and 2,000 rpm on a ThermoMixer (Eppendorf). RNA was extracted from the TRIzol mixture using Direct-zol (Zymo) columns following the manufacturer’s protocol, eluting in 90 μl. Genomic DNA was removed from purified RNA by adding 2 μl of Turbo DNase (Invitrogen) in a 100-μl final volume using the provided buffer and incubating 30 min at 37°C. DNase reaction products were cleaned up by bringing to a 200-μl volume using water and vortexing with 200 μl acid–phenol-chloroform–isoamyl alcohol (IAA) (Invitrogen). After centrifugation, the top layer was extracted and ethanol precipitated with 20 μl 3 M sodium acetate (NaOAc), 2 μl GlycoBlue (Invitrogen), and 600 μl ice-cold ethanol. After incubation, samples were spun at maximum speed for 30 min at 4°C, washed twice with ice-cold 70% ethanol, and resuspended in water.

### Preparation of ligation RNA-seq libraries.

With the exception of MazF data, which was from an earlier study where RNA was extracted from cells expressing MazF for 5 min using a similar bulk culture protocol (19), ligation libraries were prepared as described below. Cells were grown on plates and in overnight cultures as described above with the addition of 0.4% glucose to prevent leaky expression of the toxin from the arabinose inducible promoter. Cultures were back-diluted to an OD_600_ of 0.02 in 1.5 ml of M9 medium with added glucose and grown to an OD of 0.2 to 0.35 in 24-well plates. Cultures were pelleted by spinning for 5 min on a benchtop centrifuge at 4,000 × *g*. Pellets were washed once with M9 medium lacking glucose, respun, and resuspended in M9 medium lacking glucose. On a fresh 24-well plate, individual cultures were back-diluted to an OD of 0.1 and returned to the plate reader. After 30 min of growth, toxin was induced by adding arabinose to 0.2%, and cells were returned to the plate reader. After 10 min, 1 ml of the culture was removed and used to extract RNA as described above. Ligation RNA-seq libraries were prepared as described previously (19).

### Preparation of template switch RNA-seq libraries.

Cell cultures were grown and RNA was prepared as described for ligation RNA-seq libraries above. RNA was extracted from samples either 5 min or 30 min after arabinose induction for all toxins. To deplete rRNA from the 5-min samples, we used a previously described home brew rRNA depletion kit as a base protocol and modified it to work in a small-volume 96-well format (33). We first prepared a 100 μM (total molarity, not per oligonucleotide) oligonucleotide mix of the 19 Gram-negative oligonucleotides and 2 E. coli 5S rRNA-specific oligonucleotides. Using the calculator and extended protocol (https://github.com/peterculviner/ribodeplete) to calculate volumes and prepare master mixes, we prepared reaction mixtures with a 15-μl total volume, an oligonucleotide/RNA ratio of 3, and a bead/oligonucleotide ratio of 10. Before preparation of depletion reactions, streptavidin magnetic beads (21 μl per reaction; NEB) were washed once in an equal volume of 1× SSC (1× SSC is 0.15 M NaCl plus 0.015 M sodium citrate) and then resuspended in 7.5 μl 1× SSC per reaction with 1 μl per reaction of Superase-In (Invitrogen). The bead-inhibitor mixture was prepared in bulk, aliquoted into individual wells of a 96-well plate, and left at room temperature. Next, we prepared depletion reaction mixtures. A single depletion reaction mixture had 250 ng of input RNA in a 7.5-μl final volume with 1× SSC, 1 mM EDTA, and 0.1 μl oligonucleotide mix. Oligonucleotides were annealed to the rRNA by placing the depletion reaction mixtures on a thermocycler set at 70°C for 5 min followed by a gradual (0.1°C/s) ramp down to 25°C. Using a multichannel pipette, the annealing reaction mixtures were added to the 96-well plate and pipetted up and down 20 times to mix. After 5 min incubation at room temperature, a plastic cover was placed on the plate, and it was incubated for 5 min at 50°C on a thermocycler. The plate was then removed from the thermocycler and placed on a magnetic rack. Immediately after the reactions clarified (∼30 s), a multichannel pipette was used to save 11 μl of supernatant, being careful to not disturb the pellets. The supernatant containing the rRNA-depleted sample was saved in strip tubes at −80°C. We used supernatant directly to construct libraries without further purification.

Next, we constructed libraries (extended step-by-step protocol available at https://github.com/peterculviner/endoribonucmap). Both rRNA-depleted samples and total RNA samples were inputted into the first reverse transcription reaction. For fragmented libraries, 3.75 μl of RNA sample was added to a strip tube containing 1.5 μl of 10× reaction buffer (10× buffer is 500 mM Tris [pH 8], 750 mM KCl, 120 mM MgCl_2_) and 1.5 μl of 25 μM reverse transcription primer (since primers were barcoded, a different primer was assigned to each reaction). Fragmentation was accomplished by incubating this high-magnesium mixture at 95°C for 3 min on a ThermoMixer and returning it to ice. Fragmentation reaction mixtures were changed into 15-μl reverse transcription reaction mixtures by adding 0.75 μl 10 mM deoxynucleoside triphosphates (dNTPs), 0.3 μl 100 mM dCTP, 3 μl 100 mM DTT, 3 μl 5 M betaine, 0.375 μl Maxima H Minus reverse transcriptase (Thermo), 0.75 μl Superase-In (Invitrogen), and 0.075 μl water. To facilitate the large number of reactions, we added these reagents in the form of a master mix and conducted reactions in a 96-well plate. For unfragmented libraries, we followed the above protocol but did not heat reaction mixtures after addition of 10× reaction buffer and reverse transcription primer. Reverse transcription reaction mixtures were placed on a thermocycler with the following program: 10 min at 25°C, 50 min at 50°C, and 5 min at 85°C. Plates were saved at −80°C after reactions were complete.

Next, reaction mixtures with unique barcodes (up to 24 mixtures) were combined into a single pool for the rest of the protocol. To ensure that we achieved approximately equal numbers of reads in our final sequencing run, we conducted the rest of the protocol once using an equal input (2 μl) from each reverse transcription reaction. With this equal pool, we conducted a small-scale MiSeq run to determine the contribution of each sample to the final pool; from these read counts, we calculated the correct amount of each reverse transcription reaction to add to achieve equal numbers of reads from all reactions.

After combining uniquely barcoded reaction mixtures into pools, we brought the volume of each pool to 190 μl with water. To this mixture, we added 10 μl of resuspended AMPure XP beads (Beckman). Resuspended beads were prepared by pelleting 50 μl of magnetic beads into a tube, pelleting them on a magnetic rack, and resuspending them in 10 μl 10 mM Tris (pH 8). To the 200-μl sample/bead mixture, we added 200 μl of 20% (wt/vol) polyethylene glycol (PEG) 8000–2.5 M NaCl. The reactions were allowed to precipitate for 5 min at room temperature before being placed on a magnetic rack to pellet. After the tubes clarified (∼5 min), we carefully removed the supernatant and washed the pellet twice with 400 μl of fresh 80% ethanol. After removing all ethanol by a quick spin on a benchtop centrifuge, the pellets were allowed to dry at room temperature. After the pellets had dried, we resuspended each pellet in 10 μl of 10 mM Tris (pH 8)–0.1 mM EDTA by pipetting after removal from the magnetic rack. After incubation of the tubes for 5 min off the rack, the samples were returned to the rack and the supernatant was transferred to a fresh tube, with care taken not to pipette up any magnetic beads.

These pools were next subjected to a second round of reverse transcription to add the 3′ amplification region via template switching. To do this, the 10 μl was added to a strip tube with 4 μl of stock 5× Maxima H Minus reverse transcriptase reaction buffer, 0.5 μl of 100 μM template switching oligonucleotide, 1 μl of 10 mM dNTPs, 0.4 μl of 100 mM dCTP, 1 μl of Superase-In (Invitrogen), 0.5 μl of Maxima H Minus reverse transcriptase (Thermo), and 2.6 μl of water for a final reaction volume of 20 μl. Reaction mixtures were placed on a thermocycler with the following program: 10 min at 25°C, 30 min at 42°C, 5 min at 85°C. Following this reaction, samples were stored at −20°C or purified using the AMPure XP bead-based protocol as described after the first reverse transcription reaction, with the exception that reactions were instead resuspended in 20 μl of 10 mM Tris (pH 8)–0.1 mM EDTA.

To identify the correct number of cycles to amplify libraries, we conducted 10-μl trial qPCRs on each pool. Trial qPCRs were prepared by mixing 5 μl 2× iTaq universal SYBR green Supermix (Bio-Rad), 0.2 μl of any of the i7 amplification primers (100 μM stock), 0.2 μl of the i5 amplification primer (100 μM stock), 3 μl of preamplification library, and 2.6 μl of water. Reaction mixtures were loaded onto a 384-well plate and run on a Viia 7 qPCR machine using the following parameters: 95°C for 5 min and then 25 cycles of 98°C for 30 s and 60°C for 45 s. The cycle number where the amplification plot broke approximately one-half the maximum signal was used for the final PCR. For the final PCR, we prepared 30-μl reaction mixtures using a 15-μl 2× KAPA HiFi HotStart ReadyMix PCR kit (Kapa), 0.6 μl of i7 primer (using a unique i7 primer barcode for each pool; 100 μM stock), 0.6 μl of i5 primer (100 μM stock), 9 μl of preamplification library, and 4.8 μl of water. PCRs were placed on a thermocycler with the following program: 98°C for 45 s, 11 to 16 cycles of 98°C for 15 s, 60°C for 30 s, and 72°C for 30 s, and then 72°C for 60 s. The number of amplification cycles for each pool was determined by the trial qPCR described above. After completion, PCRs were brought to a 200 μl volume with water and size selected with AMPure XP beads using the following protocol. For each reaction, 50 μl of beads was pelleted on a magnetic rack and resuspended in 100 μl of 20% PEG 8000 (wt/vol)–2.5 M NaCl. This mixture was added to each reaction mixture and allowed to incubate for 5 min at room temperature before being returned to the magnetic rack. After the solution clarified, the supernatant (300 μl) was transferred to a fresh tube and the bead pellet (with high-molecular-weight [MW] products) was discarded. Next, 50 μl of beads was pelleted on a magnetic rack and resuspended in 40 μl of 20% PEG 8000 (wt/vol)–2.5 M NaCl. This mixture was added to the supernatant to achieve a 340-μl final volume and was allowed to incubate for 5 min at room temperature before being returned to the magnetic rack. After the solution clarified, the supernatant (containing low-MW products such as primer dimers) was discarded and the pellet was washed twice with 80% ethanol as described for previous bead purifications. The sample was resuspended in 11 μl of 10 mM Tris (pH 8). Libraries were sequenced on either a MiSeq or NovaSeq instrument using a custom read 1 primer. Primers for sequencing library preparation are shown in Table S2.

10.1128/mBio.02012-21.5 TABLE S2 Primers. Download Table S2, XLSX file, 0.01 MB. Copyright © 2021 Culviner et al. 2021 Culviner et al. https://creativecommons.org/licenses/by/4.0/ This content is distributed under the terms of the Creative Commons Attribution 4.0 International license.

### Modifications to library protocol for tRNA sequencing.

To enable capture of low-MW species, such as tRNA, we used the protocol described above except for modifying the reaction cleanups (i) between the first and second reverse transcription steps and (ii) after the second reverse transcription step. For the first cleanup, we pooled the tRNA samples as described above but ran the pool on a 10% Tris-borate-EDTA (TBE)–urea gel (Invitrogen) cutting just above a primer dimer band present in a no-template control up to ∼150 nucleotides (nt) by comparison to a double-stranded-DNA (dsDNA) ladder. The gel region was crushed and soaked in 400 μl of TE at 70°C for 10 min with vortexing. To remove solids from the gel slurry, the mixture was spun through a 0.22 Spin-X filter column (Corning). Small nucleic acids were next precipitated onto AMPure XP beads by resuspending a 100-μl AMPure XP bead pellet in the sample solution and adding 4 parts 1 M guanidium HCl in 100% ethanol for each part sample. After incubating 5 min at room temperature, samples were loaded on a magnetic rack and allowed to clarify (∼1 min). After removing the supernatant, the bead pellets were washed twice with 500 μl 80% ethanol and dried and resuspended in 10 μl as described in the standard bead purification protocol above. For the second cleanup, we modified the AMPure XP bead purification protocol to allow smaller nucleic acids to precipitate. To do this, we resuspended a 50-μl AMPure XP bead pellet in 30 μl of 10 mM Tris (pH 8) and added the 20-μl second reverse transcription reaction mixture, 90 μl of 20% PEG 8000 (wt/vol)–2.5 M NaCl, and 60 μl of 100% isopropanol. After allowing this to incubate at room temperature for 5 min, we placed it on the magnetic rack and proceeded with the rest of the standard bead purification protocol (washes, drying, and resuspension in 20 μl 10 mM Tris [pH 8]).

### Isotopic labeling of mature and nascent rRNA.

Cells containing both toxin and antitoxin vectors were grown in M9 medium with a 40-ml final culture volume with a pulse of 5 μCi of [5,6-^3^H]uridine and a 1,000-fold excess chase of cold uridine at the indicated times. Cells were harvested by centrifugation at 10,000 × *g* for 1 min at 4°C. Cell pellets were washed with once with lysis buffer (20 mM Tris [pH 8], 100 mM NH_4_Cl, 10 mM MgCl_2_, 0.5 mM EDTA, and 6 mM β-mercaptoethanol), then respun, and resuspended in 300 μl of lysis buffer. To lyse cells, we added 1 μl of Ready-Lyse (Epicenter), 5 μl of Superase-In (Invitrogen), and 2 μl of Turbo DNase (Invitrogen) and incubated the lysis reaction mixture on a ThermoMixer at 6°C and 500 rpm for 5 min. To further promote lysis, we then freeze-thawed cells through 3 cycles of 10 min at −80°C and 15 min at 6°C and 500 rpm. To remove cellular debris, we then centrifuged lysate for 20 min at maximum speed and 4°C on a benchtop centrifuge. The supernatant was loaded onto a 5-to-20% linear sucrose gradient generated on a Gradient Master (BioComp) instrument in a buffer of 20 mM Tris [pH 8.0], 100 mM NH_4_Cl, and 10 mM MgCl. Samples were centrifuged in an SW41 rotor at 35,000 rpm for 4 h. Gradients were fractionated using the Gradient Master instrument with continuous monitoring of *A*_260_. One hundred microliters of each fraction was added to 4 ml of Ecoscint H (National Diagnostics), and ^3^H counts per minute were measured on a Tri-Carb 4910 TR liquid scintillation counter (PerkinElmer). Measured count-per-minute values were normalized to the volumes of each fraction and to a measurement of count-per-minute quenching across a sucrose gradient standard.

### RelE family phylogeny.

To determine the evolutionary relationship between members of the RelE family, a phylogenetic tree of toxin protein sequences was generated. Homologs for each toxin were identified with individual HMMER searches (*e* value cutoff = 0.01). Resulting sequences were pooled, and highly similar sequences were eliminated with CD-HIT (0.7 cutoff; word length = 4) (34). The resulting 1,144 sequences were aligned using MUSCLE, and a tree was built using FastTree (35, 36). The final tree was pruned to show only E. coli sequences.

### Sequencing data analysis.

FASTQ files for each barcode had adapters removed using cutadapt and were mapped to the MG1655 genome (U00096.3) using bowtie2 using the –very-sensitive argument set (37, 38). For template switch libraries, inline barcodes were also clipped from read 2 and sorted into barcode-specific FASTQ files using cutadapt. Read densities were calculated by identifying the 5′ and 3′ ends of each paired end fragment and adding one count position for all positions aligning to and between the paired reads. For 5′-end sequencing, a count was added only at the 5′ end of read 1. Counts were sequencing depth normalized by calculating size factors based on the total counts mapped to the genome with or without rRNA regions in non-rRNA-depleted and rRNA-depleted samples, respectively. Further analyses are described above, and all were accomplished with custom python code and libraries.

### Data availability.

FASTQ files for ligation libraries and template switching libraries are available on GEO. Code used to generate figures and conduct analyses is available at https://github.com/peterculviner/endoribonucmap. Sequencing data are available at GSE179607 (template switch libraries), GSE144029 (ligation libraries for all toxins except MazF), and GSE107327 (MazF ligation libraries).
