# Supplementary figures and images for: Global Analysis of the Specificities and Targets of Endoribonucleases from Escherichia coli Toxin-Antitoxin Systems
Source: mBio. 2021 Sep 21;12(5):e02012-21. doi: 10.1128/mBio.02012-21 (PMC8546651; doi:10.1128/mBio.02012-21)

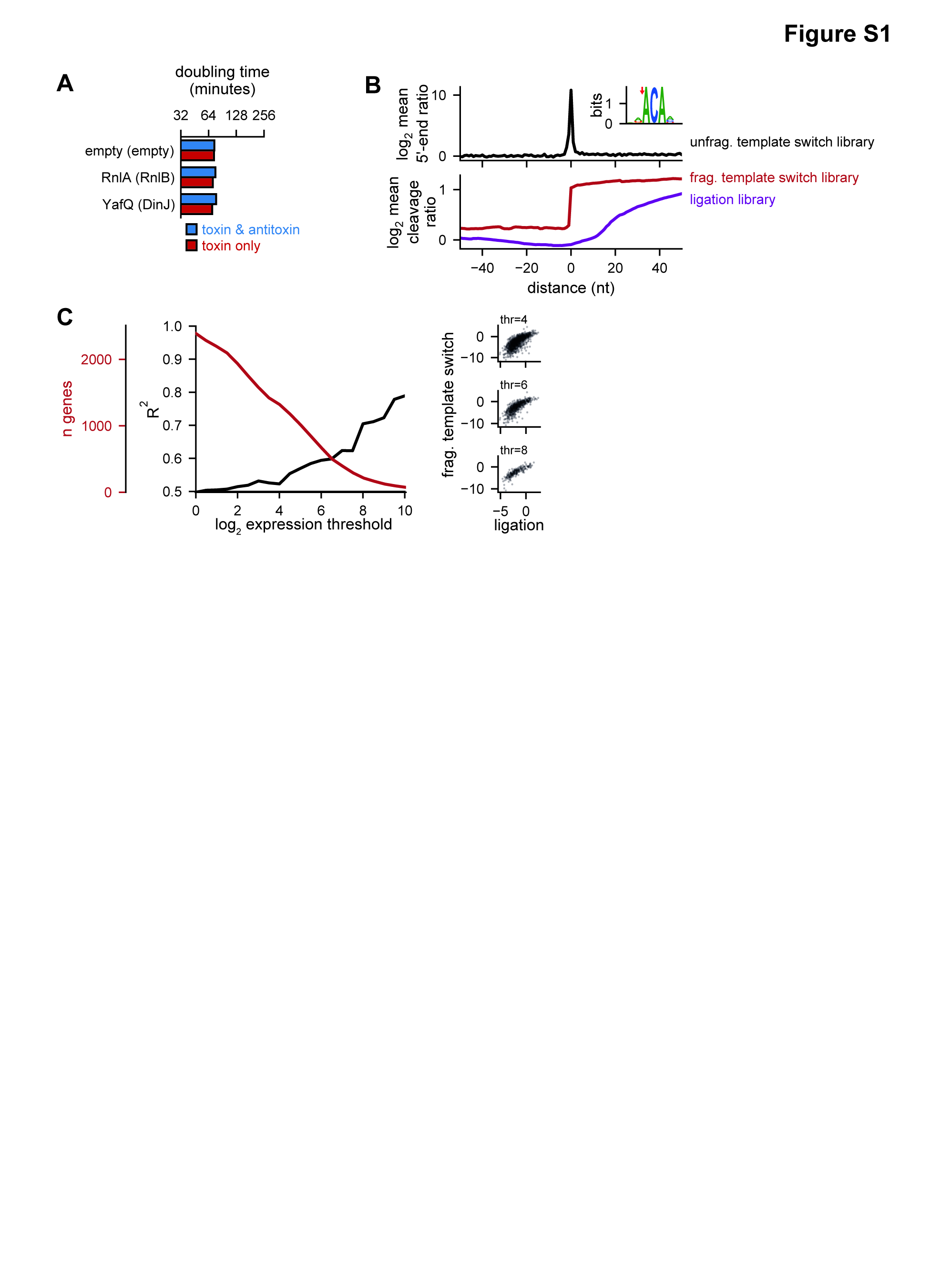

Supplement: FIG S1 [file mbio.02012-21-sf001.tif]

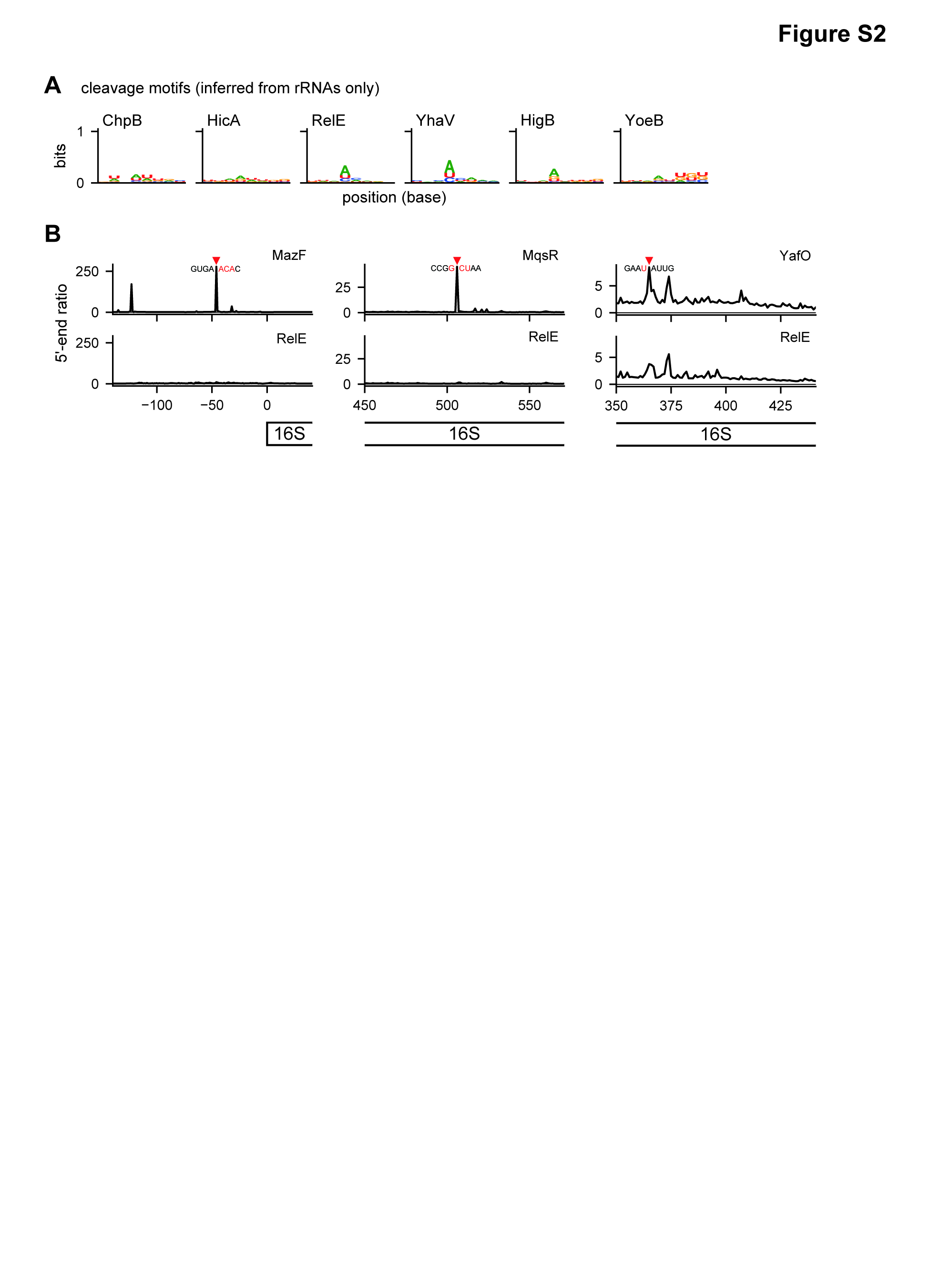

Supplement: FIG S2 [file mbio.02012-21-sf002.tif]

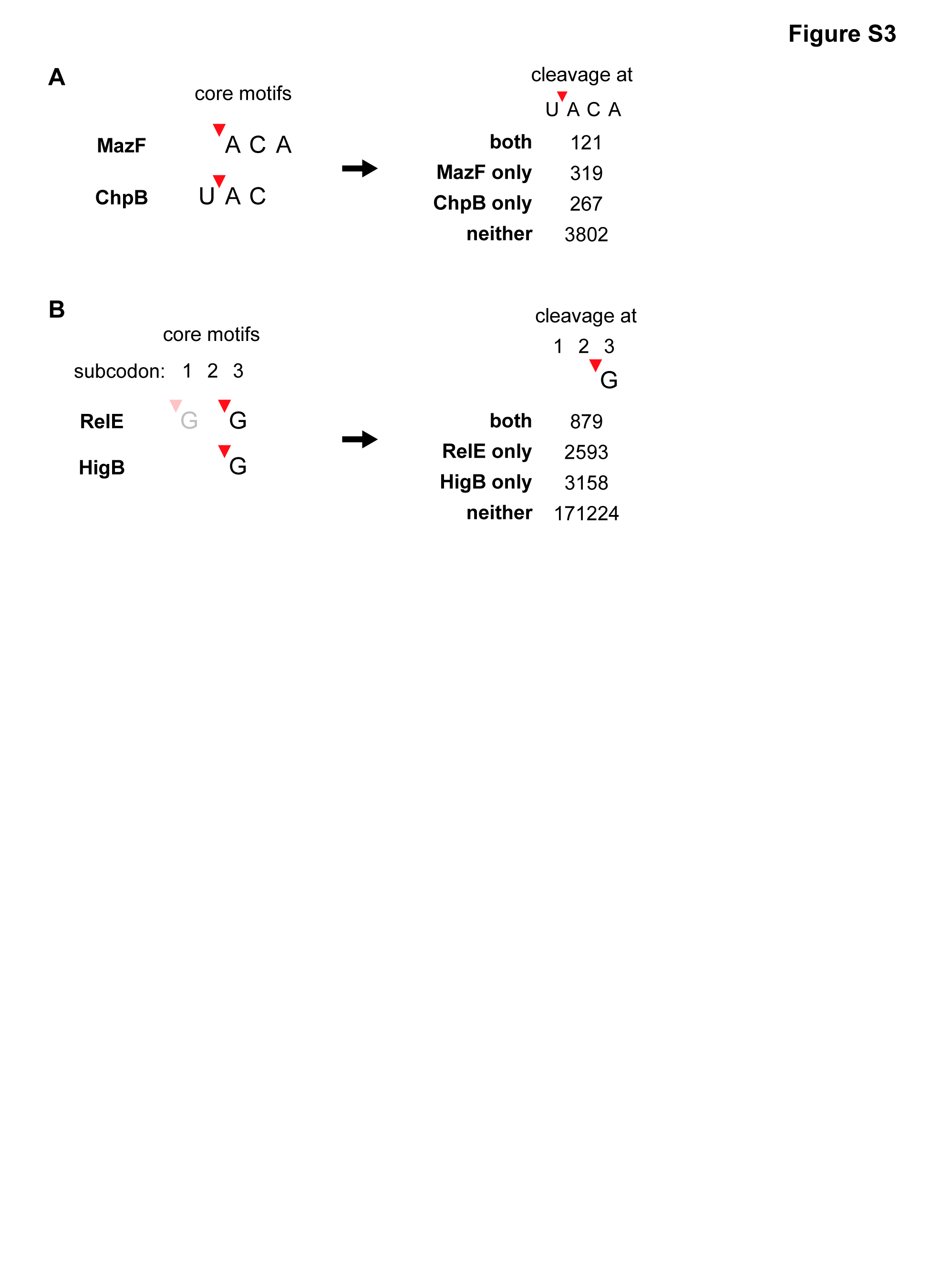

Supplement: FIG S3 [file mbio.02012-21-sf003.tif]
