# Supplementary material for: Global Analysis of the Specificities and Targets of Endoribonucleases from Escherichia coli Toxin-Antitoxin Systems
Source: mBio. 2021 Sep 21;12(5):e02012-21. doi: 10.1128/mBio.02012-21 (PMC8546651; doi:10.1128/mBio.02012-21)
Supplement: TABLE S1 [file mbio.02012-21-st001.docx]

# Table S1. Strains

| **Name** | **Genotype** | **Source** |
| --- | --- | --- |
| ML3554 | MG1655 pBAD30 | This study |
| ML3555 | MG1655 pBAD30-mazF | This study |
| ML3556 | MG1655 pBAD30-chpB | This study |
| ML3557 | MG1655 pBAD30-hicA | This study |
| ML3558 | MG1655 pBAD30-yhaV | This study |
| ML3559 | MG1655 pBAD30-mqsR | This study |
| ML3560 | MG1655 pBAD30-rnlA | This study |
| ML3561 | MG1655 pBAD30-relE | This study |
| ML3562 | MG1655 pBAD30-yoeB | This study |
| ML3563 | MG1655 pBAD30-yafO | This study |
| ML3564 | MG1655 pBAD30-yafQ | This study |
| ML3565 | MG1655 pBAD30-higB | This study |
| ML3566 | MG1655 pBAD30 pKVS45 | This study |
| ML3567 | MG1655 pBAD30-mazF pKVS45-mazE | This study |
| ML3568 | MG1655 pBAD30-chpB pKVS45-chpS | This study |
| ML3569 | MG1655 pBAD30-relE pKVS45-relB | This study |
| ML3570 | MG1655 pBAD30-yoeB pKVS45-yefM | This study |
| ML3571 | MG1655 pBAD30-yafO pKVS45-yafN | This study |
| ML3572 | MG1655 pBAD30-mqsR pKVS45-mqsA | This study |
| ML3573 | MG1655 pBAD30-higB pKVS45-higA | This study |
| ML3574 | MG1655 pBAD30-hicA pKVS45-hicB | This study |
| ML3575 | MG1655 pBAD30-yhaV pKVS45-prlF | This study |
